# Recommendations of digital health applications to patients by Portuguese General Practitioners: a cross-sectional study

**DOI:** 10.1080/13814788.2025.2567457

**Published:** 2025-11-10

**Authors:** Tânia Matias Mendes, Elena Lammila-Escalera, Cristina Jácome, Ana Luísa Neves

**Affiliations:** ^a^ RISE-Health, MEDCIDS- Department of Community Medicine, Information and Health Decision Sciences, Faculty of Medicine of the University of Porto, Porto, Portugal; ^b^ USF Cynthia, Unidade Local de Saúde Amadora-Sintra, Sintra, Portugal; ^c^ Department of Primary Care and Public Health, Imperial College London, London, UK

**Keywords:** Digital health applications, digital health, mHealth, mHealth recommendation, general practitioner

## Abstract

**Background:**

The growing adoption of digital health applications (apps) presents new opportunities for General Practitioners (GPs) to enhance care and empower patients. However, little is known about how Portuguese GPs incorporate these apps into their practice.

**Objectives:**

To identify the distinguishing characteristics of Portuguese GPs recommending the use of digital health apps to patients, and to investigate the facilitators and barriers influencing this behaviour.

**Methods:**

A cross-sectional study was conducted using an online questionnaire distributed to Portuguese GPs between July 2023 and January 2024. Univariate logistic regressions identified predictors of app recommendation. Wilcoxon rank-sum tests compared facilitators and barriers between groups.

**Results:**

A total of 126 GPs responded (72.2% women; median age 36 years [IQR: 31.8–43.0]); 45.2% recommended digital health apps. The most common were for apps for physical activity (32.4%), nutrition (21.3%), and chronic disease management (21.3%). Among GPs recommending apps, 70.2% did 1–4 times monthly. Most GPs believed that apps could improve chronic disease self-management (97.6%) and reduce face-to-face consultations (74.6%). GPs recommending apps were more likely to personally use health and fitness apps (OR 3.03), clinical decision apps (OR 3.79), and to believe that apps reduce face-to-face consultations (OR 3.46). GPs not recommending apps more often rated scientific validity as ‘very important’ (84.1% vs 61.4%, *p* = 0.006).

**Conclusion:**

Nearly half of Portuguese GPs surveyed recommended digital apps, highlighting their potential to support self-management and reduce face-to-face consultation. Broader adoption may depend on fostering greater physician confidence in app use by strengthening the scientific evidence of apps.

## Introduction

In 2011, the World Health Organisation defined mHealth as ‘medical and public health practices supported by mobile devices, such as cell phones, patient monitoring devices, personal digital assistants (PDAs) and other wireless devices’ [1]. Since then, the development of tablets, smartphones, and wearable devices has led to the proliferation of digital applications as a key resource for telemedicine. By the end of 2022, 41,517 health applications (apps) were available on the App Store^®^ and 54,546 on the Google Play Store^®^ [2].

Digital health apps are mobile software programs that help users process and manage health-related data. They are designed for anyone looking to monitor, improve, or maintain personal or community health [3]. In several countries, digital health apps available from the app stores have also been adopted at the governmental level to enhance patient health literacy, health maintenance and chronic disease management capabilities [4–6]. For example, in Portugal, the Shared Services of the Ministry of Health (SPMS) launched the SNS24® mobile app, which allows users to access a range of digital services and information from the National Health Service (NHS) [7]. Since there is no list of officially approved digital health apps, Portuguese General Practitioners (GPs) cannot formally prescribe these apps but can only suggest them to patients. Given their essential role in guiding and encouraging patients to adopt health-related behaviours, GPs can leverage these apps to support and track the maintenance of healthy habits. To achieve this, GPs must know how to choose mobile apps that best suit their patients’ goals [8].

In chronic disease management, digital health apps facilitate the remote monitoring of conditions and strengthen the relationship between the doctor and the patient [9–13]. Similarly, their utility extends to mental health, where a systematic review by Sin et al. demonstrated their effectiveness in treating common mental conditions [14]. The European Association for the Study of Diabetes and the American Diabetes Association have even collaborated to draft safety and validation guidelines for diabetes-related applications [12]. Evidence further supports the effectiveness of digital health tools in driving behaviour changes. Afshin et al. concluded that these applications can improve physical activity levels and help users reduce weight [15]. When digital interventions utilise behaviour change techniques, like prompts and goal setting, they become more effective [16]. Additionally, integrating digital health apps with medical appointments has proven more effective for maintaining healthy behaviours than using each tool alone [17]. These emerging tools have redefined Primary Care by motivating GPs to promote shared health management with patients and to encourage patients to adopt health-related behaviours that improve how they manage their conditions [18].

Preliminary evidence from Australia and Germany suggests that while GPs do use health apps themselves, they are often hesitant to recommend them to patients due to a lack of knowledge or trust in these tools [19,20]. Key factors influencing their willingness to recommend apps include perceived clinical value, concerns about risk and responsibility and familiarity with using digital tools [21,22]. However, the body of research in this area regarding the facilitators and barriers that influence recommendations is generally scarce. This leaves a significant gap in knowledge regarding how GPs in other countries, like Portugal, approach digital health apps and what factors affect their behaviours.

Consequently, this study aimed to identify the distinguishing characteristics among Portuguese GPs who recommend digital health apps and those who do not, investigating the factors influencing their recommendation behaviour.

## Methods

### Study design

This online cross-sectional survey study was conducted among GPs from Portugal. The study is described per the STrengthening the Reporting of OBservational studies in Epidemiology (STROBE) guidelines for observational studies [23]. This study was approved by the Ethics Committee of the Faculty of Medicine of the University of Porto (101/CEFMUP/2023). At the beginning of the questionnaire, participants were presented with an explanation of the study, its objectives, the expected response time, a guarantee of confidentiality, and the option to withdraw from any section of the questionnaire. The process of filling out the questionnaire began with electronically consenting to accept the terms, with a positive response needed before the first question. If the answer was negative, the questionnaire ended for the participant.

### Study participants

The sample was selected using convenience sampling. No *a priori* power calculation was conducted, as the study was exploratory. The primary aim was to collect as much data within feasibility constraints, rather than to achieve a predetermined sample size based on power considerations. Eligibility criteria included being a general and family medicine specialist or resident and practising medicine in Portugal. The organisational structure of general practice in Portugal is based on primary health care centres that operate under a chain of command. Family health units (FHU), categorised as Type A and B (with Type B having greater autonomy), are self-organised multi-professional teams that include GPs, nurses, managers and other professionals. These teams have the autonomy to establish their working processes and to negotiate performance goals with local health authorities [24]. This survey was designed to ensure the representation of physicians’ perceptions regardless of their general practice setting. Therefore, the type of the FHU did not influence participant recruitment.

The study was disseminated on social media platforms (on content pages aimed at doctors) and sent via institutional email to medical groups/communities. The Research Department of the Portuguese Association of General and Family Medicine promoted the dissemination of the study on its official communication channels, which were aimed at its members (General and Family Medicine specialists and residents).

### Data collection

Data were collected through an online questionnaire implemented on the Google Forms platform (Appendix A), which was active from July 2023 to January 2024.

The questionnaire was designed after a bibliographical review of works on the same topic of study, and the questions were comprised of four sections (Table 1). To ensure the clarity and the relevance of the instrument, we asked three to five GPs to review the questionnaire and provide informal feedback.

**Table 1.** Questionnaire sections and their descriptions.

Sections of the questionnaire	Details
1	Demographic (i.e. gender, age, academic qualifications) and professional characteristics (i.e. years of medical practice, region where they practice, medical practice in urban or rural areas, organisational model in which they operate (Family Health Units: Type A, B or private practice), experience as General Practice trainer/academic supervisor).
2	Use of digital health apps (i.e. for personal use, clinical decision-making and recommendation to patients).
3	Recommendation of digital applications (i.e. type and frequency of recommendation).
4	Factors influencing the recommendation of apps and the ability to change clinical practice (i.e. facilitators and barriers to the recommendation of applications, ability to manage chronic disease by users and potential for reducing face-to-face consultations using applications). This is assessed using a five-point scale: ‘not important’, ‘slightly important’, ‘reasonably important’, ‘important’ and ‘very important’.

### Data analysis

Descriptive statistics were used to characterise the sample, app use, and factors influencing recommendation. The sample was categorised into two groups, according to the self-reported recommendation of apps (i.e. those who recommend and those who do not). The normality of continuous variables was tested using the Kolmogorov-Smirnov test, and the median and interquartile range were used for descriptive purposes for variables without normal distribution. Absolute and relative frequencies were used for categorical variables. Univariate logistic regression was used to explore associations between participants’ characteristics and the recommendation of apps. The selection of variables was informed by a set of predefined hypotheses developed prior to analysis. These included the assumption that factors such as GPs’ personal or professional use of health technologies, clinical experience, and perceptions of app utility may influence their recommendation behaviours. Differences in the median scores for facilitators and barriers to app recommendation between GPs who do and do not recommend apps were assessed using Wilcoxon rank-sum tests with continuity correction, appropriate for the ordinal nature of the data. Statistical data analysis was performed using the features of the IBM SPSS Statistics version 29.0 (IBM Corporation®, Armonk, NY, USA) program.

## Results

### General practitioners’ characteristics

Of the 126 respondents (Table 2), the majority were women (72.2%, *n* = 91) with a median age of 36 [IQR: 31.75–43.0]. Nearly half (48.4%, *n* = 61) had 5 to 15 years of medical practice. Most participants were affiliated with Type B FHUs (52.4%, *n* = 66), which are located in predominantly urban areas (66.7%, *n* = 84). Additionally, one-third of the family doctors were internship advisors (66.7%, *n* = 84). Geographically, the South region had the highest proportion of respondents (51.6%, *n* = 65).

**Table 2.** Participants’ characteristics (*n* = 126).

Groups	Total (*n* = 126)	%
Sex		
Female	91	(72.2)
Male	35	(27.8)
Age (yrs), median [IQR]	36	[31.8, 43]
Academic degree		
Degree/Integrated master	78	(61.9)
Master/PhD	48	(38.1)
Clinical practice experience		
<5 years	31	(24.6)
5-15 years	61	(48.4)
16-25 years	18	(14.3)
26-35 years	11	(8.7)
36-45 years	5	(4.0)
Experience as GP trainer Region of the primary care centre	42	(33.3)
North	34	(27.0)
Centre	26	(20.6)
South	65	(51.6)
Urban/Rural Status of the primary care centre		
Urban	84	(66.7)
Rural	42	(33.3)
Type of primary care centre		
General Primary Health Care Centres	32	(25.4)
FHU A	26	(20.6)
FHU B	66	(52.4)
Private practice	10	(7.9)

Abbreviations: n: Number; yrs: Years; IQR: Interquartile range; PhD: Doctor of Philosophy; GP: General Practitioner; FHU: Family health units. Data on region and type of primary care centre were missing for 1 and 2 responses, respectively.

**Table 3.** The use and perception of using applications among GPs.

	GPs recommending apps (*n* = 57)		GPs not recommending apps (*n* = 69)		Total (*n* = 126)	
	n	%	n	%	n	%
Use of applications						
Use of health and fitness mobile apps	46	(80.7)	40	(58.0)	86	(68.3)
Use of clinical apps	54	(94.7)	57	(82.6)	111	(88.1)
Perceptions of using applications						
Perceived improvement of chronic disease self-management due to the use of apps	57	(100)	66	(95.7)	123	(97.6)
Perceived reduction of face-to-face consultations due to the use of apps	51	(89.5)	66	(95.7)	117	(92.9)

Abbreviations: GPs: General Practitioners; n: Number.

### Recommendation and use of applications

Most GPs reported using digital wellness and health applications in their personal lives (68.3%, *n* = 86) and to support their clinical practice and decision-making (88.1%, *n* = 111) (Table 3). A greater proportion of GPs who reported recommending apps also reported using health and fitness mobile apps (80.7%, *n* = 46) compared to those who did not recommend apps (58.0%, *n* = 40). Similarly, the use of clinical apps was more common among those who recommend (94.7%, *n* = 54), than among those who do not (82.6%, *n* = 57). The vast majority of physicians (97.6%, *n* = 123) believed that digital health applications could improve patients’ ability to self-manage chronic conditions. Furthermore, 92.9% (*n* = 117) indicated that these applications have the potential to reduce the frequency of face-to-face consultations over time.

Almost half of respondents (45.2%, *n* = 57) indicated they recommend health apps to patients. Physicians who recommended apps did so 1 to 4 times per month (70.2%, *n* = 40). The recommended applications included those focused on physical activity (77.2%, *n* = 44), nutrition (21.3%, *n* = 29) and chronic disease management e.g. for the management of Asthma, Chronic Obstructive Pulmonary Disease, Diabetes or Hypertension (21.3%, *n* = 29) (Table 4).

**Table 4.** Frequency and type of health applications recommended (*n* = 57).

	n (%)	
Frequency of recommendation		
<1 time per month	9	(15.8)
1 time per month	20	(35.1)
2 to 4 times per month	20	(35.1)
>4 times per month	8	(14.0)
Type of apps recommended		
Physical activity	44	(32.4)
Nutrition	29	(21.3)
Chronic disease management	29	(21.3)
Mental health	23	(16.9)
Menstrual cycle/contraception	4	(2.9)
Smoking	2	(1.4)
Travel	1	(0.74)
Obesity	1	(0.74)
Drugs dose/price	2	(1.4)
NHS	1	(0.74)

### Factors associated with app recommendation

Several factors were associated with a higher likelihood of recommending digital health apps (Table 5). GPs were more likely to recommend apps if they had personally used health and fitness apps (OR = 3.03; 95% CI 1.34–6.84) or clinical decision apps (OR = 3.79; 95% CI 1.01–14.17) Additionally, GPs who believed digital health apps could reduce face-to-face consultations had increased odds of recommending them (OR = 3.46; 95% CI 1.02–11.70).

**Table 5.** Univariate analysis to explain the recommendation of digital health apps (*n* = 126).

Characteristics	Crude OR	(95% CI)
Sex		
Male	Reference	
Female	0.71	(0.32–1.55)
Age	1.03	(0.99–1.07)
Academic degree		
Degree/Integrated Master	Reference	
Master/Doctorate	0.82	(0.13–5.05)
Clinical practice experience (years)		
<5	Reference	
5–15	0.49	(0.20–1.22)
16–25	0.48	(0.15–1.57)
26–35	0.57	(0.14–2.33)
36–45	0.32	(0.05–2.21)
Region		
North	Reference	
Centre	0.68	(0.23–1.99)
South	0.68	(0.29–1.60)
Urban/Rural Status		
Urban	Reference	
Rural	1.16	(0.55–2.43)
Type of primary care centre		
General primary care centre	Reference	
FHU A	0.89	(0.30–2.65)
FHU B	1.49	(0.63–3.55)
Internship supervisor		
No	Reference	
Yes	1.54	(0.73–3.24)
Use of health and fitness mobile apps		
No	Reference	
Yes	3.03	(1.34–6.84)*
Use of clinical decision apps		
No	Reference	
Yes	3.79	(1.01–14.17)*
Improving chronic disease self-management		
No	Reference	
Yes	a	
Potential reduction of face-to-face consultations due to the use of apps		
No	Reference	
Yes	3.46	(1.02–11.70)*

Abbreviations: OR: Odds ratio; CI: Confidence interval; FHU: Family Health Unit; Reference: the category used as reference (i.e. to which other categories are compared), a; insufficient number of cases. *p < 0.05.

### Facilitators and barriers influencing app recommendation

#### Facilitators

Factors influencing the decision to recommend digital health apps are detailed in (Table 6). The factors most commonly rated as ‘important’ and ‘very important’ included the app’s ease of use for patients (97.7%), patient access to necessary hardware (96.8%), and whether the app was free to use (95.2%). Other highly rated factors were patient access to the internet (95.3%), along with the GPs knowledge of digital health (93.7%) and interest in digital health (93.6%). Those who do not recommend health apps were significant more likely to rate proven scientific validity as “very important” than those who do (84.1% vs 61.4%, *p* = 0.006). Ratings for all other facilitators did not differ significantly between the two groups (Appendix B).

**Table 6.** Factors that influence the recommendation of applications.

Faciliators of recommendation	GPs recommending apps (*n* = 57)			GPs not recommending apps (*n* = 69)		Total (*n* = 126)	
	n		(%)	n	(%)	n	%
**Doctor’s knowledge of available applications**							
Very important	37		(64.9)	43	(62.3)	80	(63.5)
Important	18		(31.6)	20	(29.0)	38	(30.2)
Reasonably important	2		(3.5)	6	(8.7)	8	(6.3)
Slightly important	0		(0.0)	0	(0.0)	0	(0.0)
Not important	0		(0.0)	0	(0.0)	0	(0.0)
*Wilcoxon’s W (p-value) *	2051.5 (p = 0.625)						
**Doctor’s interest in digital health**							
Very important	32	(56.1)		44	(63.8)	76	(60. $\dot{3}$ )
Important	21	(36.8)		21	(30.4)	42	(33. $\dot{3}$ )
Reasonably important	4	(7.0)		4	(5.8)	8	(6. $\dot{3}$ )
Slightly important	0	(0.0)		0	(0.0)	0	(0.0)
Not important	0	(0.0)		0	(0.0)	0	(0.0)
*Wilcoxon’s W (p-value) *	1816.5 (p = 0.395)						
**Time available during appointments**							
Very important	33	(57.9)		48	(0.0)	81	(64.3)
Important	20	(35.1)		16	(1.4)	36	(28.6)
Reasonably important	3	(5.3)		4	(9.5)	7	(5.6)
Slightly important	1	(4.8)		1	(23.2)	2	(1.6)
Important	0	(0.0)		0	(69.6)	0	(0.0)
*Wilcoxon’s W (p-value) *	1754.5 (p = 0.219)						
**Application in Portuguese**							
Very important	30	(52.6)		39	(56.5)	69	(54.3)
Important	22	(38.6)		21	(30.4)	43	(34.1)
Reasonably important	5	(8.8)		8	(11.6)	13	(10.3)
Slightly important	0	(0.0)		1	(1.4)	1	(0.8)
Not important	0	(0.0)		(0.0)	(0.0)	0	(0.0)
*Wilcoxon’s W (p-value)*	1939.0 (p = 0.882)						
**The application is free**							
Very important	39	(68.4)		49	(71.0)	88	(69.8)
Important	16	(28.1)		16	(23.2)	32	(25.4)
Reasonably important	2	(3.5)		4	(5.8)	6	(4.8)
Slightly important	0	(0.0)		0	(0.0)	0	(0.0)
Not important	0	(0.0)		0	(0.0)	0	(0.0)
*Wilcoxon’s W (p-value)*	1931.5.0 (p = 0.833)						
**Ease of use by patient**							
Very important	46	(80.7)		54	(78.3)	100	(79.4)
Important	11	(19.3)		12	(17.4)	23	(18.3)
Reasonably important	0	(0.0)		3	(4.3)	3	(2.4)
Slightly important	0	(0.0)		0	(0.0)	0	(0.0)
Not important	0	(0.0)		0	(0.0)	0	(0.0)
*Wilcoxon’s W (p-value)*	2031.0 (p = 0.655)						
**Patient access to hardware**							
Very important	40	(70.2)		51	(73.9)	91	(72.2)
Important	15	(26.3)		16	(23.2)	31	(24.6)
Reasonably important	2	(3.5)		2	(2.9)	4	(3.2)
Slightly important	0	(0.0)		0	(0.0)	0	(0.0)
Not important	0	(0.0)		0	(0.0)	0	(0.0)
*Wilcoxon’s W (p-value)*	1892.0 (p = 0.642)						
**Patient access to the internet**							
Very important	40	(70.2)		46	(66.7)	86	(68.3)
Important	17	(29.8)		17	(24.6)	34	(27.0)
Reasonably important	0	(0.0)		6	(8.7)	6	(4.8)
Slightly important	0	(0.0)		0	(0.0)	0	(0.0)
Not important	0	(0.0)		0	(0.0)	0	(0.0)
*Wilcoxon’s W (p-value)*	2086.5 (p = 0.472)						
**Patient’s health literacy**							
Very important	25	(43.9)		33	(47.8)	58	(46.0)
Important	24	(42.1)		22	(31.9)	46	(36.5)
Reasonably important	8	(14.0)		12	(17.4)	20	(15.9)
Slightly important	0	(0.0)		1	(1.4)	1	(0.8)
Not important	0	(0.0)		1	(1.4)	1	(0.8)
*Wilcoxon’s W (p-value)*	1976.5 (p = 0.960)						
**Patient’s digital literacy**							
Very important	30	(52.6)		31	(44.9)	61	(48.4)
Important	24	(42.1)		32	(46.4)	56	(44.4)
Reasonably important	3	(5.3)		6	(8.7)	9	(7.1)
Slightly important	0	(0.0)		0	(0.0)	0	(0.0)
Not important	0	(0.0)		0	(0.0)	0	(0.0)
*Wilcoxon’s W (p-value)*	2142.0 (p = 0.337)						
**Patient’s interest in applications**							
Very important	34	(59.6)		35	(50.7)	69	(54.8)
Important	19	(33.3)		28	(40.6)	47	(37.3)
Reasonably important	4	(7.0)		5	(7.2)	9	(7.1)
Slightly important	0	(0.0)		0	(0.0)	0	(0.0)
Not important	0	(0.0)		1	(1.4)	1	(0.8)
*Wilcoxon’s W (p-value)*	2145.0 (p = 0.3243)						
**Proven scientific validity**							
Very important	35	(61.4)		58	(84.1)	93	(73.8)
Important	14	(24.6)		6	(8.7)	20	(15.9)
Reasonably important	7	(12.3)		4	(5.8)	11	(8.7)
Slightly important	1	(1.8)		1	(1.4)	2	(1.6)
Not important	0	(0.0)		0	(0.0)	0	(0.0)
*Wilcoxon’s W (p-value)*	**1533.5 (p = 0.006)**						
**Online data security**							
Very important	38	(66.7)		53	(76.8)	91	(72.2)
Important	10	(17.5)		6	(8.7)	16	(12.7)
Reasonably important	7	(12.3)		9	(13.0)	16	(12.7)
Slightly important	2	(3.5)		1	(1.4)	3	(2.4)
Not important	0	(0.0)		0	(0.0)	0	(0.0)
*Wilcoxon’s W (p-value)*	1784.5 (p = 0.2583)						
**Ease of access/extraction of data by the doctor**							
Very important	25	(43.9)		32	(46.4)	57	(45.2)
Important	16	(28.1)		21	(30.4)	37	(29.4)
Reasonably important	10	(17.5)		11	(15.9)	21	(16.7)
Slightly important	4	(7.0)		4	(5.8)	8	(6.3)
Not important	2	(3.5)		1	(1.4)	3	(2.4)
*Wilcoxon’s W (p-value)*	1867.0 (p = 0.6044)						

Abbreviations: n: Number. p-value in bold; p<0.05.

### Barriers

Several barriers to recommending apps were also identified (Table 7). The patient’s low digital literacy (87.3%), patient interest in apps (85.7%) and a lack of patient hardware access (81.8%) were cited as ‘important’ and ‘very important’, alongside a lack of patient internet access (81.0%) and low health literacy (78.6%). Ratings for all the barriers to recommendation did not differ significantly between those who recommend digital apps and those who do not.

**Table 7.** Barriers that influence the recommendation of applications.

	GPs recommending apps (*n* = 57)		GPs not recommending apps (*n* = 69)		Total (*n* = 126)	
Barriers to recommendation	n	(%)	n	(%)	n	(%)
**Lack of patient’s internet access**						
Very important	30	(52.6)	40	(58.0)	70	(55.6)
Important	17	(29.8)	15	(21.7)	32	(25.4)
Reasonably important	9	(15.8)	6	(8.7)	15	(11.9)
Slightly important	1	(1.8)	7	(10.1)	8	(6.3)
Not important	0	(0.0)	1	(1.4)	1	(0.8)
*Wilcoxon’s W (p-value)*	1939.0 (p = 0.883)					
**Lack of patient’s hardware access**						
Very important	30	(52.6)	40	(58.0)	70	(55.6)
Important	17	(29.8)	15	(23.2)	32	(25.2)
Reasonably important	7	(12.3)	7	(10.1)	14	(11.1)
Slightly important	3	(5.3)	5	(7.2)	8	(6.3)
Not important	0	(0.0)	1	(1.4)	1	(0.8)
*Wilcoxon’s W (p-value)*	1904.0 (p = 0.736)					
**Patient’s interest in applications**						
Very important	26	(45.6)	38	(55.1)	64	(50.8)
Important	22	(38.6)	22	(31.9)	44	(34.9)
Reasonably important	8	(14.0)	6	(8.7)	14	(11.1)
Slightly important	0	(0.0)	3	(4.3)	3	(2.4)
Not important	1	(1.8)	0	(0.0)	1	(0.8)
*Wilcoxon’s W (p-value)*	1788.0 (p = 0.337)					
**Patient’s low health literacy**						
Very important	27	(47.4)	29	(42.0)	55	(43.7)
Important	19	(33.3)	25	(36.2)	44	(34.9)
Reasonably important	9	(47.4)	11	(15.9)	20	(15.9)
Slightly important	1	(1.8)	4	(5.8)	5	(4.0)
Not important	1	(1.8)	0	(0.0)	1	(0.8)
*Wilcoxon’s W (p-value)*	2081.5 (p = 0.546)					
**Patient’s low digital literacy**						
Very important	30	(52.6)	37	(53.6)	67	(53.2)
Important	18	(34.6)	25	(36.2)	43	(34.1)
Reasonably important	8	(14.0)	5	(7.2)	13	(10.3)
Slightly important	0	(0.0)	2	(2.9)	2	(1.6)
Not important	1	(1.8)	0	(0.0)	1	(0.8)
*Wilcoxon’s W (p-value)*	1902.0 (p = 0.727)					
**Concern about data confidentiality**						
Very important	12	(21.1)	21	(30.4)	33	(26.2)
Important	16	(28.1)	17	(24.6)	33	(26.2)
Reasonably important	16	(28.1)	17	(24.6)	33	(26.2)
Slightly important	9	(15.8)	10	(14.5)	19	(15.1)
Not important	4	(7.0)	4	(5.8)	3	(6.3)
*Wilcoxon’s W (p-value)*	1783.0 (p = 0.356)					
**Possibility of patient’s wrong self-management/self-medication**						
Very important	16	(28.1)	19	(27.5)	35	(27.6)
Important	21	(36.8)	25	(36.2)	46	(36.5)
Reasonably important	13	(22.8)	19	(27.5)	32	(25.4)
Slightly important	5	(8.8)	3	(4.3)	8	(6.3)
Not important	2	(3.5)	3	(4.3)	5	(4.0)
*Wilcoxon’s W (p-value)*	1966.5 (p = 1.0)					
**Lack of doctor’s training in application recommendation**						
Very important	26	(45.6)	36	(52.2)	62	(49.2)
Important	19	(33.3)	17	(24.6)	36	(28.6)
Reasonably important	9	(15.8)	12	(17.4)	21	(16.7)
Slightly important	2	(3.5)	3	(4.3)	5	(4.0)
Not important	1	(1.8)	1	(1.4)	2	(1.6)
*Wilcoxon’s W (p-value)*	1887.0 (p = 0.675)					

Abbreviations: n: Number. Data were missing for 1 response.

## Discussion

### Principal findings

Almost half of GPs surveyed in Portugal currently used digital health apps in clinical practice, with the most recommended apps focusing on physical activity, nutrition and chronic disease management. Doctors who use health and fitness apps are more likely to recommend them to patients. GPs who do not recommend apps report lack of scientific evidence as the most important facilitator behind their decision to recommend. No other significant differences in facilitator or barrier ratings were found between the two groups. Overall, the main factors influencing GPs’ recommendations for these apps are ease of use, patient access to free apps and compatible hardware. Additionally, a GP’s knowledge of and interest in digital health significantly influences their recommendation decisions. However, key barriers to recommendation include patients’ low digital literacy, lack of interest and limited access to necessary technology, such as hardware or internet connectivity.

### Comparison with existing literature

The findings of this study align with but also challenge patterns observed in existing literature on the recommendation of digital health applications by family physicians. Previous research reports prescription rates ranging from 10% to 50% [19,20,25], consistent with the 45% observed in this study. Notably, Wanger et al. highlight that urban practice settings have a positive impact on prescribing behaviour, as highlighted by Wanger et al. [20]. However, our findings suggest a small but non-significant increase in the likelihood of recommendation among rural physicians. While no definitive conclusions can be drawn, environmental and infrastructure factors likely play a role in digital health adoption.

Demographics and experience reveal additional variations. While Della Vecchia et al. reported that men were likelier to prescribe health applications, this study concludes that there is no strong evidence to suggest a significant difference between male and female physicians [25]. While prior research indicates that younger physicians are more likely to recommend, our findings reveal a small positive association between recommendation and age [19,20,25]. While this association is weak and inconclusive, it is likely influenced by the low variance in age among participants rather than a true lack of association. This highlights the need for further investigation with larger sample sizes to better understand this relationship. These discrepancies may reflect contextual differences that warrant exploration in future research.

Personal use of digital technologies is another key factor influencing recommendation behaviour. Evidence demonstrates that personal use of apps and electronic health records enhances digital maturity and increases the likelihood of prescribing such tools [25,26]. Our results align with these conclusions, reinforcing the importance of personal digital engagement in driving clinical adoption.

Prior literature suggests that the most common applications used by patients are for mental health, mindfulness, nutrition, physical activity, women’s health, and chronic disease management [19,27,28]. While physicians in this study generally recommended apps one to four times per month, slightly less frequently than the five times per month reported in Catalonia, the similarity in app types may reflect global trends, the suitability of apps for these health areas, and their availability in the market [27].

Key factors influencing adoption remain consistent across studies. Ease of use, scientific validity, and data security emerged as critical determinants in our research, aligning with previous studies and the results of a systematic review conducted by Gagnon et al. [29]. These factors, along with app cost, design, and approval by reputable health entities, shape practices worldwide [19,20,22,25,30].

Physicians broadly view health applications as valuable for improving therapy adherence, supporting chronic disease management, and promoting health education [5]. While opinions on their transformative potential vary, as noted by Sarradon-Eck et al. this study revealed a cautious optimism among clinicians [20,22]. Most participants recognised the long-term potential of apps to reduce the number of face-to-face consultations, with some reservations about immediate impacts. The robust evaluation of these health applications should, therefore, be encouraged, as this is essential to reassure clinicians of their effectiveness and to cultivate greater confidence in their integration into primary care. Moreover, future research is required to examine studies across different counties, providing a more reliable understanding of behaviours during this critical period of digitalisation in clinical practice.

### Strengths and limitations

To our knowledge, this is the first study to characterise the recommendation of digital health applications by Portuguese GPs, filling a critical gap in the literature. Dissemination was facilitated through collaboration with the Portuguese Association of General and Family Medicine (APMGF), a trusted organisation among GPs. Leveraging APMGF’s reach and credibility allowed the survey to target a diverse population of GPs practising across different regions and clinical settings in Portugal.

However, several limitations must be acknowledged. Convenience sampling was used, and a priori power calculation was not conducted, due to the exploratory nature and feasibility constraints of the study. These factors may affect the robustness of our findings, which should be interpreted with appropriate caution. A limitation of the study is the lack of a theoretical framework, such as the Theoretical Domains Framework, to guide the questionnaire design [31]. Integrating such a framework could have provided a more structured approach to capturing a broader range of digital health app recommendation determinants that may impact recommendation behaviour. Disseminating the survey via online platforms also introduces the potential for selection bias, as respondents who are frequent users of online technologies and digital applications may have been more likely to participate. Consequently, it is possible that the respondents have a personal interest in digital health and may have been disproportionately represented in our survey, potentially skewing the results. The survey did not differentiate between apps freely available for download and those officially approved by authorities, which could have provided additional insight into recommendation behaviour.

Furthermore, this national survey could not capture the local contextual factors, such as cultural and social influences, that could impact recommendation behaviour. The exclusion of these variables limits our ability to analyse all determinants comprehensively. Another limitation of this study is that only univariate analysis was conducted; future research would benefit from multivariate approaches to account for potential confounding factors and better isolate the independent effects of variables influencing recommendation behaviours. Plus, subsequent investigations should consider incorporating a broader range of contextual factors, such as reimbursement policies and recommendation durations, while using more representative sampling techniques to enhance the robustness and applicability of findings.

## Conclusion

Nearly half of surveyed GPs in Portugal recommend health apps to their patients, with most acknowledging these tools’ significant role in promoting self-management. Doctors who personally use health and well-being apps are more likely to suggest them to their patients. Many GPs believe that health apps have the potential to revolutionise clinical practice in the long term by reducing the need for face-to-face consultations. However, since scientific evidence emerged as a key factor for GPs who do not currently recommend apps, further efforts are required to ensure these apps undergo a comprehensive evaluation to ensure their validity. Improving transparency and access to evidence may help encourage greater adoption among this group. Additionally, to fully embrace the digital transformation of primary care, it is essential to prioritise improving digital literacy and integrating user-centred design in the development of these technologies.

## Supplementary Material

Supplemental Material
