# Supplementary material for: Recommendations of digital health applications to patients by Portuguese General Practitioners: a cross-sectional study
Source: Eur J Gen Pract. 2025 Nov 10;31(1):2567457. doi: 10.1080/13814788.2025.2567457 (PMC12604134; doi:10.1080/13814788.2025.2567457)
Supplement: Supplemental Material [file IGEN_A_2567457_SM8617.docx]

**Appendix A.**

Portuguese to English translation of the questionnaire disseminated

**Questionnaire on the Prescription Pattern of Digital Health Applications by Portuguese Family Doctors**

Section 1

I have read and understood the initial explanation about this questionnaire and the use of the collected data, and I consent to participating in this study.

○ Yes

○ No

I would like to receive the results of this study via email.

○ Yes

○ No

Section 2

Doctor’s Characteristics and Professional Practice

Gender:

○ Male

○ Female

○ Other / Prefer not to say

Age:

(Free text field)

Academic qualifications:

○ Bachelor's degree

○ Master's degree

○ Doctorate

Years of medical practice:

○ < 5 years

○ 5 to 15 years

○ 15 to 25 years

○ 25 to 35 years

○ 35 to 45 years

○ 45 to 55 years

○ ≥ 55 years

Region of practice:

○ Aveiro District

○ Beja District

○ Braga District

○ Bragança District

○ Castelo Branco District

○ Coimbra District

○ Évora District

○ Faro District

○ Guarda District

○ Leiria District

○ Lisbon District

○ Portalegre District

○ Porto District

○ Santarém District

○ Setúbal District

○ Viana do Castelo District

○ Vila Real District

○ Viseu District

○ Autonomous Region of Madeira

○ Autonomous Region of the Azores

Do you practise medicine in a:

○ Predominantly urban area

○ Predominantly rural area

Organisational model in which you work (select all that apply):

□ Personalised Healthcare Unit

□ Family Health Unit (Model A)

□ Family Health Unit (Model B)

□ Private Practice

Are you a Specialty Internship Supervisor for General and Family Medicine?

○ Yes

○ No

Section 3

Use of Applications

Do you currently use Health and Well-being Applications for personal use?

○ Yes

○ No

Do you use applications in your medical practice to support clinical decisions?

○ Yes

○ No

Do you recommend or prescribe Health and Well-being Applications to your patients?

○ Yes

○ No

Section 4

Type and Frequency of Prescribed Applications

If yes, how often do you prescribe them?

○ Less than once a month

○ Once a month

○ 2 to 4 times a month

○ More than 4 times a month

If yes, do you prescribe applications in the following areas?

□ Mental Health

□ Physical Activity

□ Nutrition

□ Chronic Disease Management by the patient (e.g., Asthma, COPD, Diabetes, Hypertension)

□ Others

Section 5

Factors Influencing the Prescription of Applications

How important do you consider each of the following factors when prescribing applications?

(1 - Not important; 2 - Slightly important; 3 - Moderately important; 4 - Important; 5 - Very important)

- Doctor’s knowledge of available applications

- Doctor’s interest in the field of digital health

- Time available during consultation

- Application available in Portuguese

- Application being free of charge

- Ease of use for the patient

- Patient’s access to hardware (e.g., smartphone)

- Patient’s access to the internet

- Patient’s good health literacy

- Patient’s good digital literacy

- Patient’s interest in using applications

- Scientifically validated effectiveness

- Online security of collected data

- Ease of access/extraction of data by the doctor

Among the following, how important do you consider each factor as a barrier to prescribing mobile applications?

(1 - Not important; 2 - Slightly important; 3 - Moderately important; 4 - Important; 5 - Very important)

- Lack of internet access for the patient

- Lack of access to hardware (e.g., smartphones)

- Low patient interest

- Poor health literacy among patients

- Poor digital literacy among patients

- Concerns about data confidentiality

- Possibility of incorrect self-management/self-medication by patients

- Lack of medical training in prescribing applications

Do you believe that the correct use of chronic disease management applications by patients can contribute to better control of their clinical condition?

○ Yes

○ No

Do you believe that the correct use of chronic disease management applications by patients can lead to a reduction in face-to-face consultations in primary healthcare?

○ No

○ Yes, in the long term

○ Yes, in the short term
